# Supplementary material for: Klebsiella pneumoniae clinical isolates with features of both multidrug-resistance and hypervirulence have unexpectedly low virulence
Source: Nat Commun. 2023 Dec 2;14:7962. doi: 10.1038/s41467-023-43802-1 (PMC10693551; doi:10.1038/s41467-023-43802-1)
Supplement: Supplementary file 1 — Supplementary Information [file 41467_2023_43802_MOESM1_ESM.pdf]

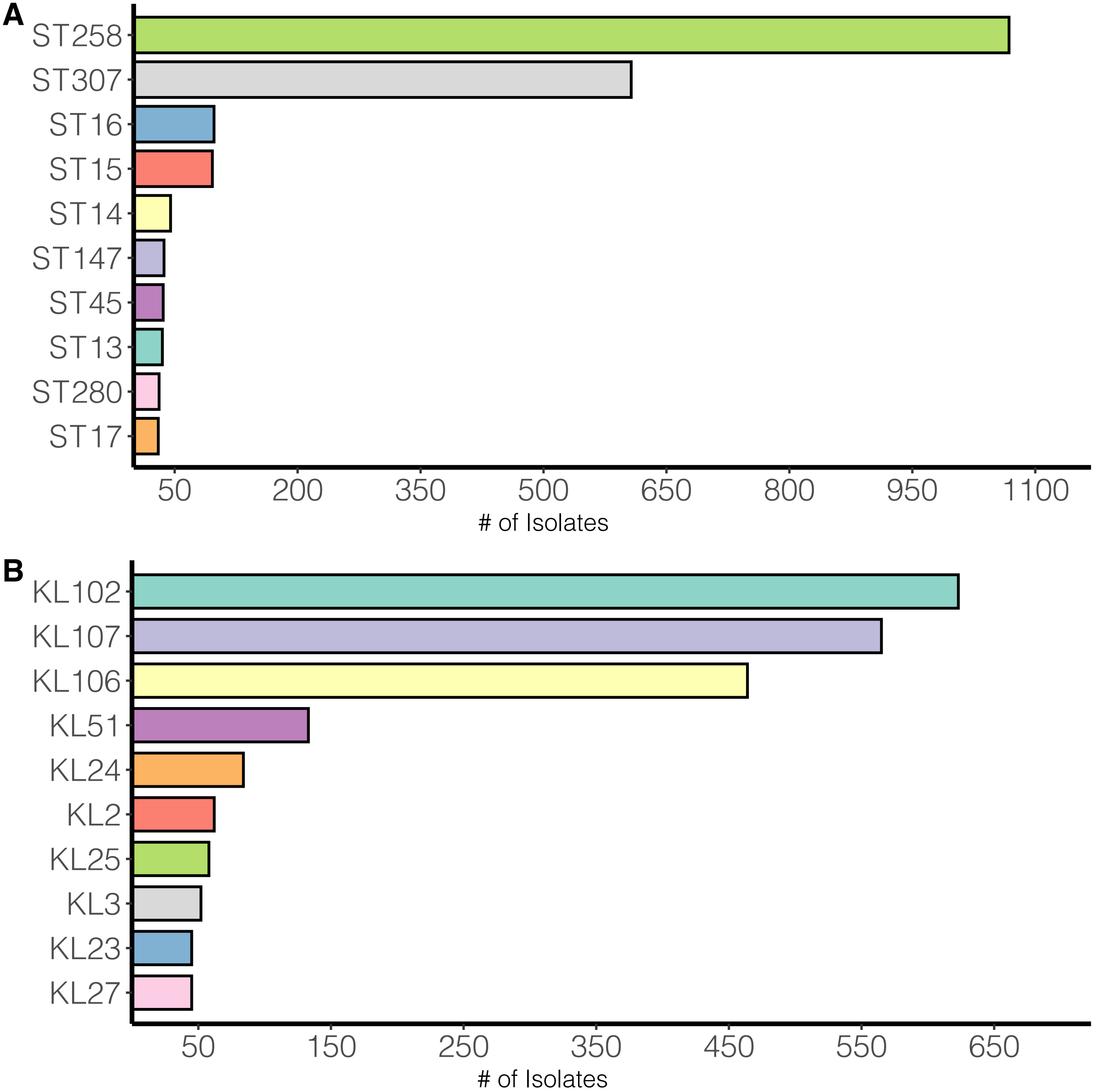

Figure S1. Sequence types and capsule loci of the 2,608 K. pneumoniae isolates screened in this study. The number of isolates in the three K. pneumoniae collections that had the most common (A) sequence types or (B) capsule loci types are shown. Source data are provided as a Source Data file.

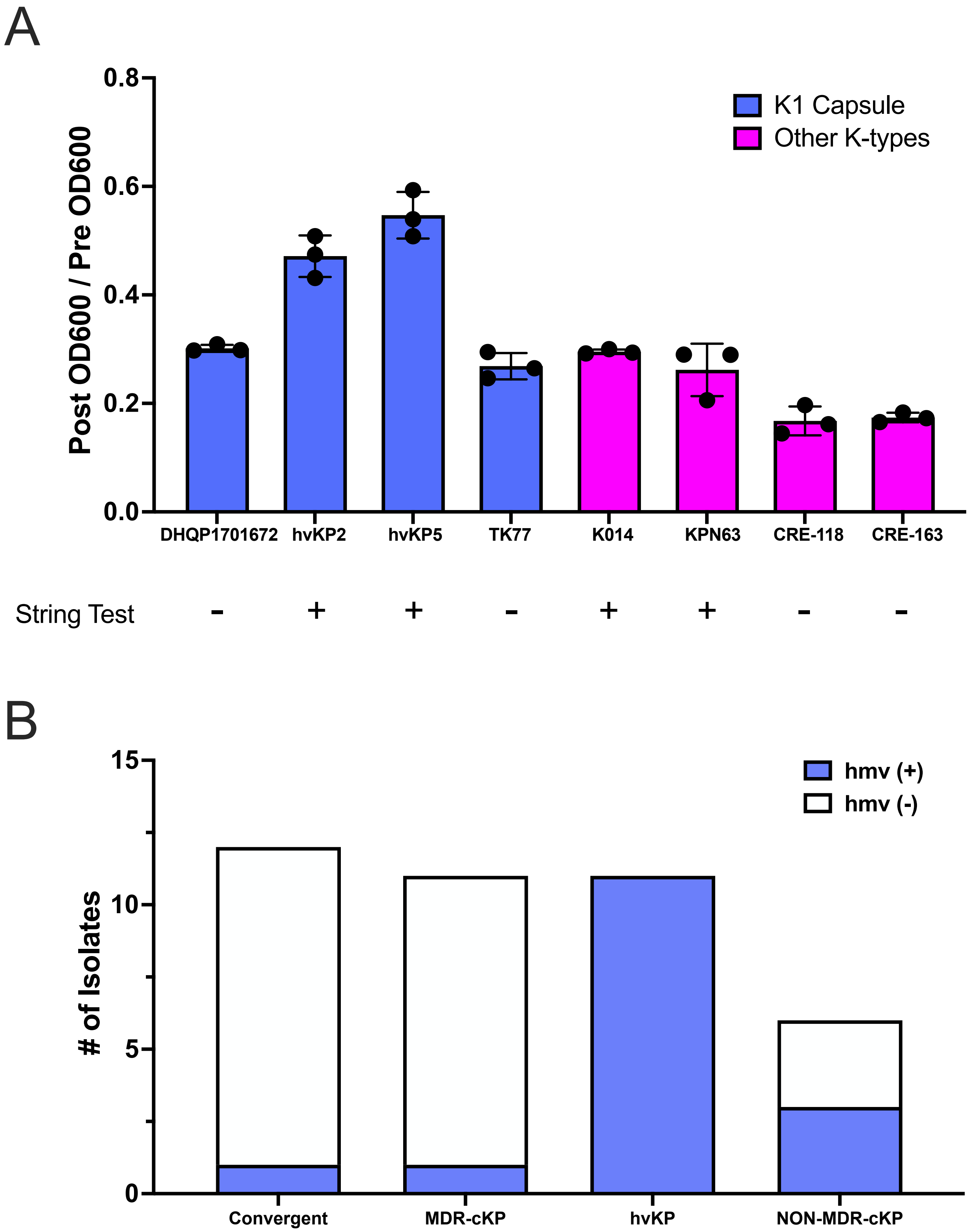

Figure S2. Hypermucoviscosity of convergent, MDR-cKP, hvKP, and NON-MDR-cKP isolates. (A) Degree of hypermucoviscosity of a subset of K1 and non-K1 isolates, as measured by resistance to sedimentation. Hypermucoviscosity was quantified by the ratio of the OD600 values for bacterial suspensions after and before centrifugation. (B) hmv colony morphology was measured by the string test. The number of hmv+ and hmv- isolates in each group is shown. Source data are provided as a Source Data file.

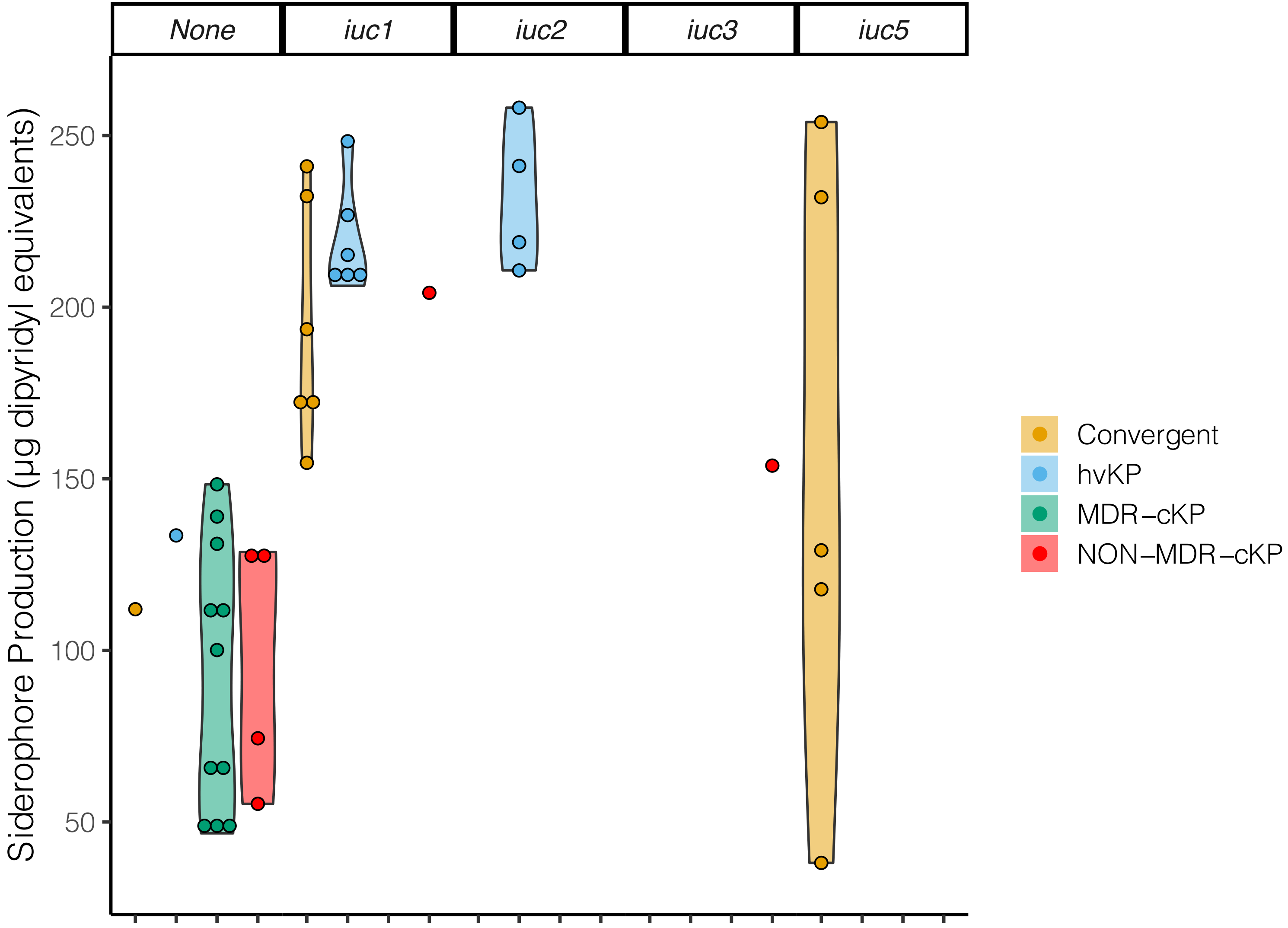

Figure S3. Siderophore production by each isolate with respect to the lineage of its aerobactin biosynthesis locus. Total siderophore production for each of the 40 convergent, MDR-cKP, NON-MDR-cKP, and hvKP is indicated. “None” indicates no iuc genes were found in the isolate. iuc1, iuc2, iuc3, and iuc5 are distinct lineages of aerobactin. Source data are provided as a Source Data file.

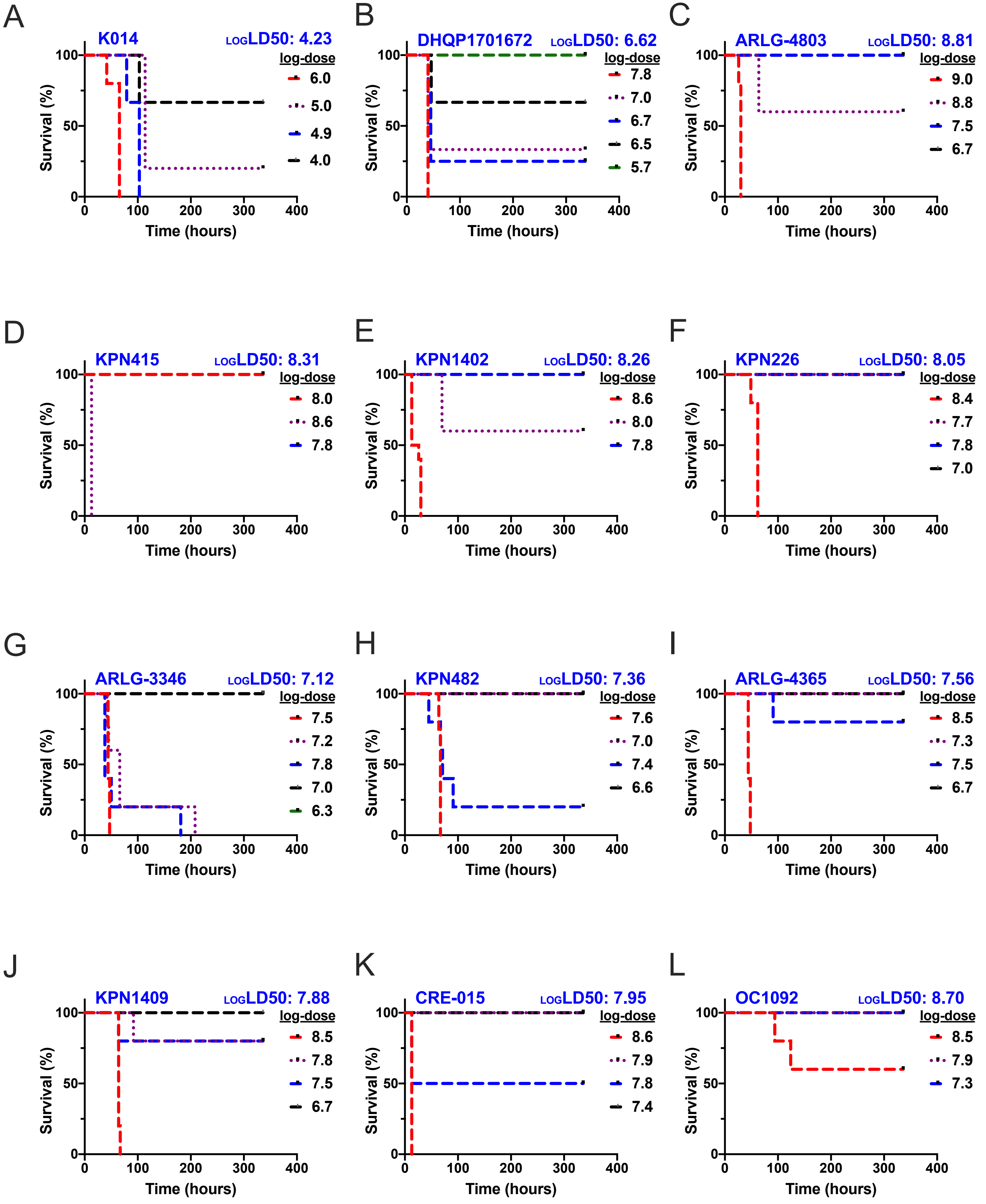

Figure S4. Survival curves of mice infected with each of the 12 representative convergent isolates. C57BL/6 mice were infected intranasally with the indicated doses for each convergent isolate: (A) K014, (B) DHQP1701672, (C) ARLG-4803, (D) KPN415, (E) KPN1402, (F) KPN226, (G) ARLG-3346, (H) KPN482, (I) ARLG-4365, (J) KPN1409, (K) CRE-015, and (L) OC1092. The total number of mice, dose, and mortalities for each group are listed in Supplementary Data 5. Log(LD50) values were determined using the r package “drc” and are labeled in blue for each graph. Source data are provided as a Source
