# Supplementary material for: Klebsiella pneumoniae clinical isolates with features of both multidrug-resistance and hypervirulence have unexpectedly low virulence
Source: Nat Commun. 2023 Dec 2;14:7962. doi: 10.1038/s41467-023-43802-1 (PMC10693551; doi:10.1038/s41467-023-43802-1)
Supplement: Supplementary file 3 — Description of Additional Supplementary Files [file 41467_2023_43802_MOESM3_ESM.pdf]

Description of Additional Supplementary files

Supplementary Data 1. Kleborate results of all isolates included in this study. Kleborate analysis of sequence type, capsule loci, virulence gene content, and antimicrobial resistance gene content of illumina assemblies of (A) all genomes included in the study and (B) all convergent isolates identified in this study.

Supplementary Data 2. Kleborate analysis of MDR, convergent, and hvKP isolates included in this study. (A) Kleborate analysis of sequence type, capsule loci, virulence gene content, and antimicrobial resistance gene content of illumina assemblies of the 40 representative isolates included in the study. (B) Phenotypic analysis of LD50, siderophore production, capsule production, serum resistance and hypermucoviscosity.

Supplementary Data 3. Kleborate analysis of plasmids identified in this study. Kleborate analysis of hybrid assemblies the 40 representative isolates included in this study including (A) all plasmids and (C) all chromosomes identified in this study. (B) Results of plasmid typing using MOB Suite.

Supplementary Data 4. Complete genomes of isolates included in this study. Complete Genomes, accession numbers, size, and identified replicons of each complete genome in this study.

Supplementary Data 5. Survival data and dosing use to calculate LD50. Dosing, mortalities, and total mice used for each strain in determining LD50.
