# Supplementary material for: Klebsiella pneumoniae clinical isolates with features of both multidrug-resistance and hypervirulence have unexpectedly low virulence
Source: Nat Commun. 2023 Dec 2;14:7962. doi: 10.1038/s41467-023-43802-1 (PMC10693551; doi:10.1038/s41467-023-43802-1)
Supplement: Supplementary file 9 — Reporting Summary [file 41467_2023_43802_MOESM9_ESM.pdf]

nature portfolio

Corresponding author(s): Travis Kochan

Last updated by author(s): Oct 26, 2023

Reporting Summary

Nature Portfolio wishes to improve the reproducibility of the work that we publish. This form provides structure for consistency and transparency in reporting. For further information on Nature Portfolio policies, see our Editorial Policies and the Editorial Policy Checklist.

Statistics

For all statistical analyses, confirm that the following items are present in the figure legend, table legend, main text, or Methods section.

n/a	Confirmed	
☐	☒	The exact sample size (n) for each experimental group/condition, given as a discrete number and unit of measurement
☐	☒	A statement on whether measurements were taken from distinct samples or whether the same sample was measured repeatedly
☐	☒	The statistical test(s) used AND whether they are one- or two-sided<br>*Only common tests should be described solely by name; describe more complex techniques in the Methods section.*
☒	☐	A description of all covariates tested
☒	☐	A description of any assumptions or corrections, such as tests of normality and adjustment for multiple comparisons
☒	☐	A full description of the statistical parameters including central tendency (e.g. means) or other basic estimates (e.g. regression coefficient) AND variation (e.g. standard deviation) or associated estimates of uncertainty (e.g. confidence intervals)
☐	☒	For null hypothesis testing, the test statistic (e.g. F, t, r) with confidence intervals, effect sizes, degrees of freedom and P value noted<br>*Give P values as exact values whenever suitable.*
☒	☐	For Bayesian analysis, information on the choice of priors and Markov chain Monte Carlo settings
☒	☐	For hierarchical and complex designs, identification of the appropriate level for tests and full reporting of outcomes
☒	☐	Estimates of effect sizes (e.g. Cohen's d, Pearson's r), indicating how they were calculated

Our web collection on statistics for biologists contains articles on many of the points above.

Software and code

Policy information about availability of computer code

Data collection	No software was used for data collection
Data analysis	Data analysis was completed using published programs: R (package 'drc'),, Kleborate v2.1, Kaptive v 0.7.3, Cytoscape v 3.8.2, MOB-suite v3.1.7, Fasttree2 v 4.0.3, bwa-0.7.15, Mashtree v 1.4.3, iTOL v4, MaSurca v4.1.0, Polypolish v0.5.0, Medaka v1.9.1, Raven v2.3, Unicycler v0.5.0, Flye v2.9, Trycyler v0.5.4, Filtlong v0.2.1, Guppy v3.4.5, SPAdes v3.9.1, Trimmomatic v0.36, and Graphpad Prism 8, Sratoolkit v2.11.1

For manuscripts utilizing custom algorithms or software that are central to the research but not yet described in published literature, software must be made available to editors and reviewers. We strongly encourage code deposition in a community repository (e.g. GitHub). See the Nature Portfolio guidelines for submitting code & software for further information.

Data

Policy information about availability of data

All manuscripts must include a data availability statement. This statement should provide the following information, where applicable:

- Accession codes, unique identifiers, or web links for publicly available datasets
- A description of any restrictions on data availability
- For clinical datasets or third party data, please ensure that the statement adheres to our policy

The whole-genome assemblies of bacteria sequenced for this study have been deposited at GenBank under BioProject number PRJNA788509. Assembly accession

numbers are included in Table S4. Kleborate data are included in Table S1. Phenotypic data for individual strains are found in Table S2. There are no restrictions on data availability. Source data are provided with this paper.

Research involving human participants, their data, or biological material

Policy information about studies with human participants or human data. See also policy information about sex, gender (identity/presentation), and sexual orientation and race, ethnicity and racism.

Reporting on sex and gender	NA
Reporting on race, ethnicity, or other socially relevant groupings	NA
Population characteristics	NA
Recruitment	NA
Ethics oversight	NA

Note that full information on the approval of the study protocol must also be provided in the manuscript.

Field-specific reporting

Please select the one below that is the best fit for your research. If you are not sure, read the appropriate sections before making your selection.

☒ Life sciences ☐ Behavioural & social sciences ☐ Ecological, evolutionary & environmental sciences

For a reference copy of the document with all sections, see nature.com/documents/nr-reporting-summary-flat.pdf

Life sciences study design

All studies must disclose on these points even when the disclosure is negative.

Sample size	No Sample size calculations were performed. In order to minimize pain and distress in animals, sample sizes were determined to be the smallest size required to get a quality LD50 measurement. This was determined to be a minimum of 15 mice for each strain. However, in most cases the sample sizes were between 20-30 mice per strain.
Data exclusions	No data were excluded
Replication	Because the goal of these experiments was to determine an LD50, specific dosing strategies were performed. Animal experiments were repeated at least twice on separate days. The first experiment usually included a larger range of dosing where the second experiment would include dosing that fell in between the range to better identify the LD50. Specific doses and number of mice for each strain are included in the supplement. All attempts at replication were successful.
Randomization	Animals used were randomly split into groups of 3-5 mice, per strain, per dose.
Blinding	Investigators were blinded for animal experiments. Strains and dosing were determined, then randomly coded throughout the experiment so that the investigators did not know which strains were associated with which animals to eliminate bias.

Reporting for specific materials, systems and methods

We require information from authors about some types of materials, experimental systems and methods used in many studies. Here, indicate whether each material, system or method listed is relevant to your study. If you are not sure if a list item applies to your research, read the appropriate section before selecting a response.

Materials & experimental systems

n/a	Involved in the study	
☒	☐	Antibodies
☒	☐	Eukaryotic cell lines
☒	☐	Palaeontology and archaeology
☐	☒	Animals and other organisms
☒	☐	Clinical data
☒	☐	Dual use research of concern
☒	☐	Plants

Methods

n/a	Involved in the study	
☒	☐	ChIP-seq
☒	☐	Flow cytometry
☒	☐	MRI-based neuroimaging

Animals and other research organisms

Policy information about studies involving animals; ARRIVE guidelines recommended for reporting animal research, and Sex and Gender in Research

Laboratory animals	6-8 week old C57BL/6 female mice were purchased from Jackson Labs. Temperatures of 65-75°F (~18-23°C) with 40-60% humidity. 12:12 light dark cycle.
Wild animals	No wild animals were used in this study.
Reporting on sex	The findings included in this manuscript include only female mice. However, it was determined previously in our lab that there are no sex based differences in outcomes of Klebsiella pneumoniae infection in our mouse model of pneumonia.
Field-collected samples	The study did not involve field collected samples
Ethics oversight	Northwestern University Animal Care and Use Committee

Note that full information on the approval of the study protocol must also be provided in the manuscript.

Plants

Seed stocks	NA
Novel plant genotypes	NA
Authentication	NA
